# Supplementary material for: Longitudinal linked-read sequencing reveals ecological and evolutionary responses of a human gut microbiome during antibiotic treatment
Source: Genome Res. 2021 Aug;31(8):1433–46. doi: 10.1101/gr.265058.120 (PMC8327913; doi:10.1101/gr.265058.120)
Supplement: Supplemental Material [file supp_31_8_1433__DC1.html]

Longitudinal linked-read sequencing reveals ecological and evolutionary responses of a human gut microbiome during antibiotic treatment — Supplemental Material 

# Longitudinal linked-read sequencing reveals ecological and evolutionary responses of a human gut microbiome during antibiotic treatment

## Supplemental Material

- Supplemental\_Data\_S1.txt
- Supplemental\_Data\_S2.txt
- Supplemental\_Data\_S3.txt
- Supplemental\_Data\_S4.txt
- Supplemental\_Data\_S5.pdf
- Supplemental\_Data\_S6.pdf
- Supplemental\_Data\_S7.txt
- Supplemental\_Materials.pdf
- Supplemental\_Code\_S1.zip
- Supplemental\_Table\_S1\_JDedit.csv
- Supplemental\_Table\_S2\_JDedit.csv
- Supplemental\_Table\_S3\_JDedit.csv
