# Supplementary material for: Longitudinal linked-read sequencing reveals ecological and evolutionary responses of a human gut microbiome during antibiotic treatment
Source: Genome Res. 2021 Aug;31(8):1433–46. doi: 10.1101/gr.265058.120 (PMC8327913; doi:10.1101/gr.265058.120)
Supplement: Supplemental Material [file supp_gr.265058.120_Supplemental_Data_S5.pdf]

Sample: 6041
Well Location: C1
Created: Thursday, July 21, 2016 2:23:11 PM

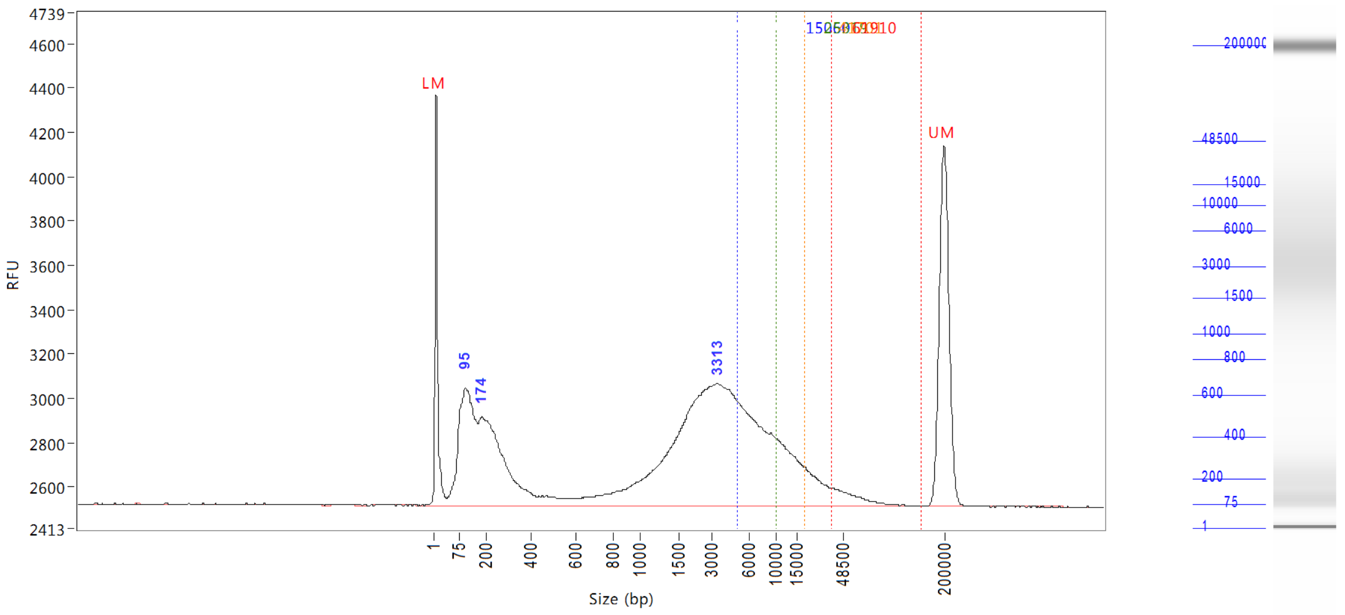

Peak	Size (bp)	Conc. (ng/uL)	From (bp)	To (bp)	Avg. Size (bp)	CV%	RFU	Corr. Peak Area
1	1 (LM)	0.0078	0	34	2	288.02	1847	15.172
2	95	0.1595	34	156	101	29.31	529	25.957
3	174	0.1744	156	435	240	26.86	397	28.385
4	3313	0.6263	656	91364	6819	149.15	547	101.953
5	200000 (UM)	0.0111	173395	240570	200080	3.49	1626	21.590

TIC:	0.9601	ng/uL
TIM:	4.720	nmole/L
Total Conc.:	0.9786	ng/uL

Smear Analysis						
	40000 bp to 165000 bp	0.0175 ng/ul	1.8 %Total	0.000 nmole/L	61910 Avg. Size (b.p.)	34.17 %CV
	20000 bp to 165000 bp	0.0441 ng/ul	4.5 %Total	0.002 nmole/L	41701 Avg. Size (b.p.)	51.62 %CV
	10000 bp to 165000 bp	0.1033 ng/ul	10.6 %Total	0.007 nmole/L	25319 Avg. Size (b.p.)	78.98 %CV
	5000 bp to 165000 bp	0.2329 ng/ul	23.8 %Total	0.025 nmole/L	15060 Avg. Size (b.p.)	107.32 %CV

Sample Peak Width (sec): 10 Sample Min Peak Height: 50 Sample Baseline V to V?: Y Sample Baseline V to V pts: 3
Sample Filter: Binomial # of Pts for Filter: 3 Sample Start Region (min): 0 Sample End Region (min): 35
Manual Baseline Start (min): 10 Manual Baseline End (min): 35
Marker Peak Width (sec): 5 Marker Min Peak Height: 500 Marker Baseline V to V?: N Marker Baseline V to V pts: 3
Lower Marker Selection: First Peak > 500 RFU Upper Marker Selection: Last Peak > 500 RFU
Ladder Size (bp): 1, 75, 200, 400, 600, 800, 1000, 1500, 3000, 6000, 10000, 15000, 48500, 200000
Quantification Using: Ladder Final Concentration (ng/uL): 0.1250 Dilution Factor: 12.0
Min. RFU for Data Processing: 3

 Copyright 2015 Advanced Analytical Technologies, Inc.

Sample: 6037
Well Location: C2
Created: Thursday, July 21, 2016 2:23:11 PM

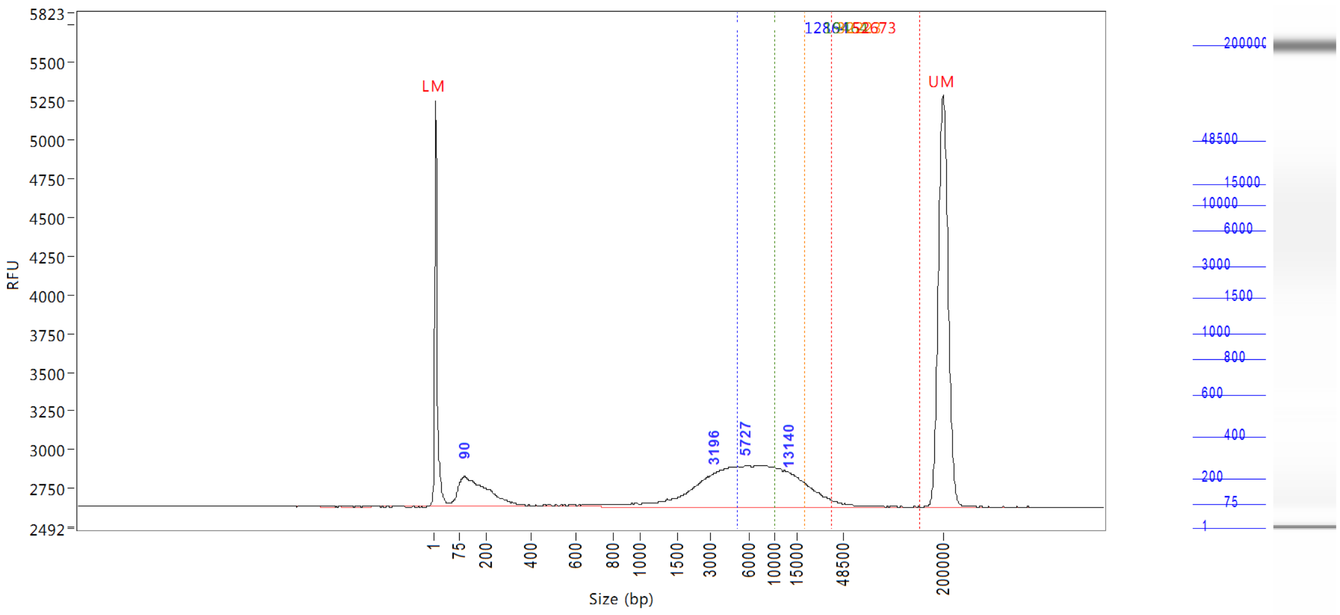

Peak	Size (bp)	Conc. (ng/uL)	From (bp)	To (bp)	Avg. Size (bp)	CV%	RFU	Corr. Peak Area
1	1 (LM)	0.0078	0	37	1	595.30	2620	20.885
2	90	0.0678	37	270	138	41.94	192	15.193
3	3196	0.0449	989	3235	2239	28.76	217	10.069
4	5727	0.1235	3235	13140	6954	38.90	266	27.674
5	13140	0.0365	13140	69933	23297	44.84	221	8.175
6	200000 (UM)	0.0132	166744	237619	200187	3.52	2657	35.394

TIC:	0.2727	ng/uL
TIM:	1.299	nmole/L
Total Conc.:	0.2850	ng/uL

Smear Analysis						
	40000 bp to 165000 bp	0.0034 ng/ul	1.2 %Total	0.000 nmole/L	52673 Avg. Size (b.p.)	27.71 %CV
	20000 bp to 165000 bp	0.0190 ng/ul	6.7 %Total	0.001 nmole/L	32223 Avg. Size (b.p.)	38.46 %CV
	10000 bp to 165000 bp	0.0574 ng/ul	20.1 %Total	0.005 nmole/L	19464 Avg. Size (b.p.)	59.43 %CV
	5000 bp to 165000 bp	0.1225 ng/ul	43.0 %Total	0.016 nmole/L	12864 Avg. Size (b.p.)	78.29 %CV

```
Sample Peak Width (sec): 10   Sample Min Peak Height: 50    Sample Baseline V to V?: Y    Sample Baseline V to V pts: 3
Sample Filter: Binomial       # of Pts for Filter: 3        Sample Start Region (min): 0  Sample End Region (min): 35
Manual Baseline Start (min): 10     Manual Baseline End (min): 35
Marker Peak Width (sec): 5    Marker Min Peak Height: 500   Marker Baseline V to V?: N    Marker Baseline V to V pts: 3
Lower Marker Selection: First Peak > 500 RFU                Upper Marker Selection: Last Peak > 500 RFU
Ladder Size (bp): 1, 75, 200, 400, 600, 800, 1000, 1500, 3000, 6000, 10000, 15000, 48500, 200000
Quantification Using: Ladder         Final Concentration (ng/uL): 0.1250          Dilution Factor: 12.0
Min. RFU for Data Processing: 3
```

 Copyright 2015 Advanced Analytical Technologies, Inc.

Sample: 4025
Well Location: C3
Created: Thursday, July 21, 2016 2:23:11 PM

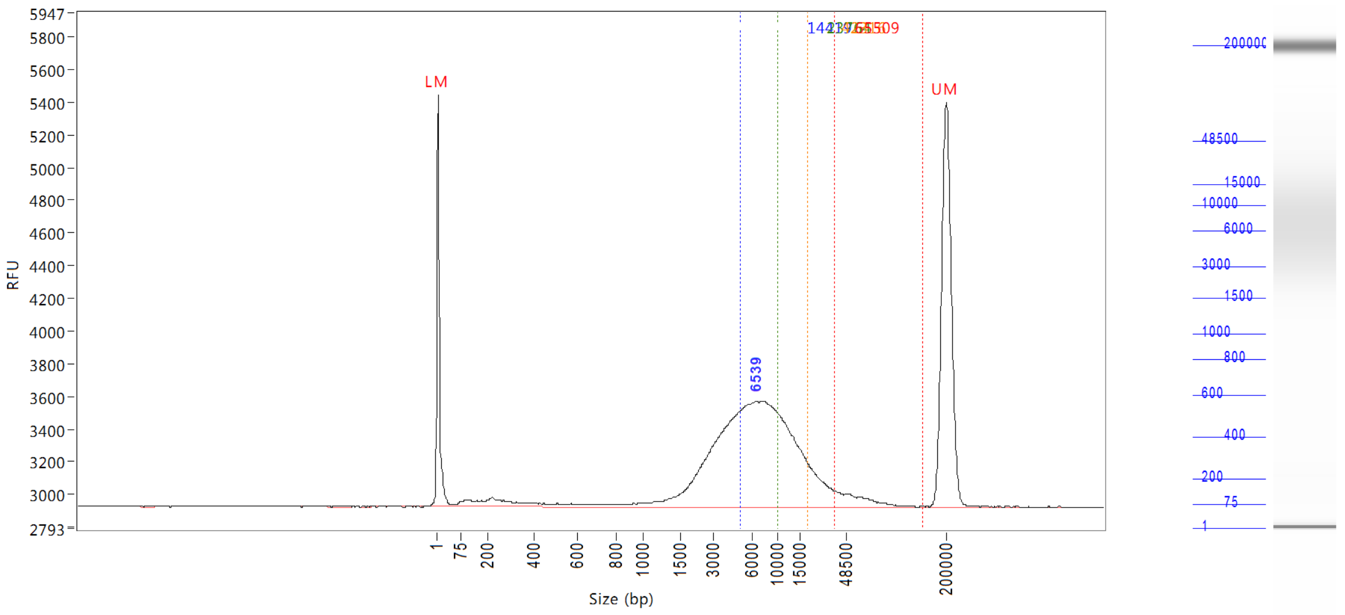

Peak	Size (bp)	Conc. (ng/uL)	From (bp)	To (bp)	Avg. Size (bp)	CV%	RFU	Corr. Peak Area
1	1 (LM)	0.0078	0	47	2	381.14	2513	20.105
2	6539	0.4291	1021	47419	8665	88.69	643	92.569
3	200000 (UM)	0.0128	168961	244258	200001	3.60	2474	33.216

TIC:	0.4291	ng/uL
TIM:	0.108	nmole/L
Total Conc.:	0.4802	ng/uL

Smear Analysis						
	40000 bp to 165000 bp	0.0177 ng/ul	3.7 %Total	0.000 nmole/L	65509 Avg. Size (b.p.)	35.71 %CV
	20000 bp to 165000 bp	0.0473 ng/ul	9.9 %Total	0.002 nmole/L	42316 Avg. Size (b.p.)	55.79 %CV
	10000 bp to 165000 bp	0.1280 ng/ul	26.7 %Total	0.009 nmole/L	23764 Avg. Size (b.p.)	84.77 %CV
	5000 bp to 165000 bp	0.2887 ng/ul	60.1 %Total	0.033 nmole/L	14419 Avg. Size (b.p.)	109.15 %CV

```
Sample Peak Width (sec): 10   Sample Min Peak Height: 50    Sample Baseline V to V?: Y    Sample Baseline V to V pts: 3
Sample Filter: Binomial       # of Pts for Filter: 3        Sample Start Region (min): 0  Sample End Region (min): 35
Manual Baseline Start (min): 10     Manual Baseline End (min): 35
Marker Peak Width (sec): 5    Marker Min Peak Height: 500   Marker Baseline V to V?: N    Marker Baseline V to V pts: 3
Lower Marker Selection: First Peak > 500 RFU                Upper Marker Selection: Last Peak > 500 RFU
Ladder Size (bp): 1, 75, 200, 400, 600, 800, 1000, 1500, 3000, 6000, 10000, 15000, 48500, 200000
Quantification Using: Ladder          Final Concentration (ng/uL): 0.1250          Dilution Factor: 12.0
Min. RFU for Data Processing: 3
```

 Copyright 2015 Advanced Analytical Technologies, Inc.

Sample: 6038.1
Well Location: C4
Created: Thursday, July 21, 2016 2:23:11 PM

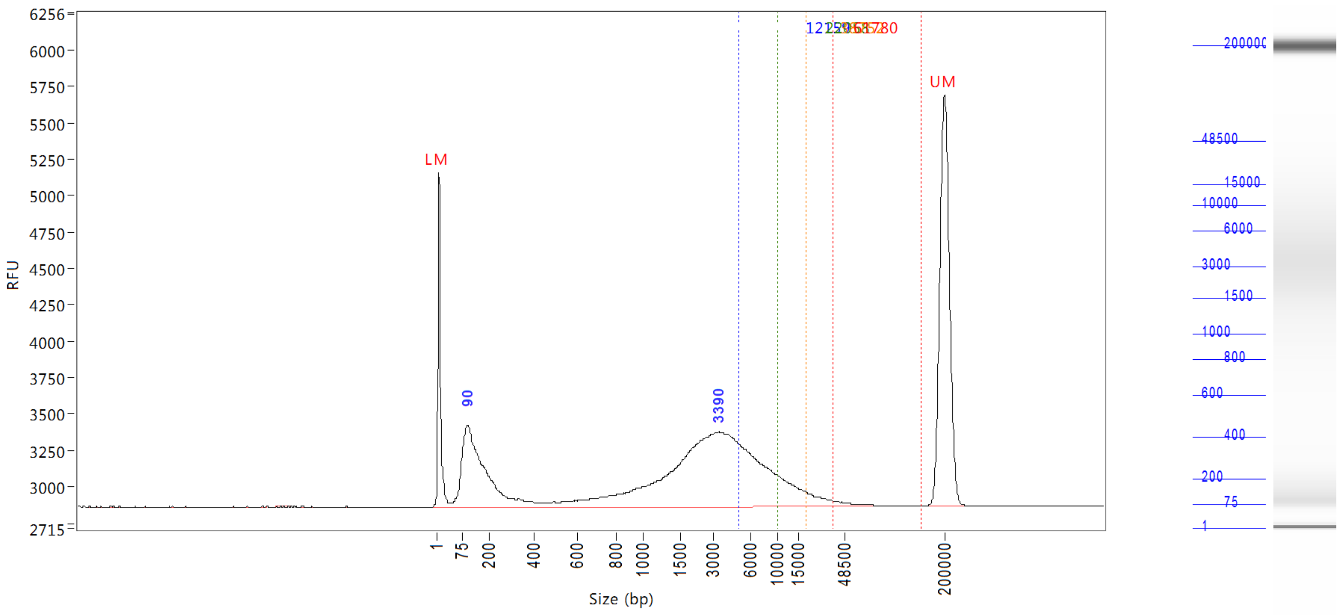

Peak	Size (bp)	Conc. (ng/uL)	From (bp)	To (bp)	Avg. Size (bp)	CV%	RFU	Corr. Peak Area
1	1 (LM)	0.0078	0	33	2	331.48	2299	18.087
2	90	0.1903	33	368	141	49.64	560	36.932
3	3390	0.4627	502	27968	4202	95.81	511	89.793
4	200000 (UM)	0.0161	174873	243521	200116	3.51	2821	37.537

TIC:	0.6530	ng/uL
TIM:	3.694	nmole/L
Total Conc.:	0.6732	ng/uL

Smear Analysis						
	40000 bp to 165000 bp	0.0054 ng/ul	0.8 %Total	0.000 nmole/L	61780 Avg. Size (b.p.)	38.74 %CV
	20000 bp to 165000 bp	0.0173 ng/ul	2.6 %Total	0.001 nmole/L	38752 Avg. Size (b.p.)	54.34 %CV
	10000 bp to 165000 bp	0.0492 ng/ul	7.3 %Total	0.004 nmole/L	22058 Avg. Size (b.p.)	79.82 %CV
	5000 bp to 165000 bp	0.1387 ng/ul	20.6 %Total	0.019 nmole/L	12159 Avg. Size (b.p.)	104.84 %CV

```
Sample Peak Width (sec): 10   Sample Min Peak Height: 50    Sample Baseline V to V?: Y    Sample Baseline V to V pts: 3
Sample Filter: Binomial       # of Pts for Filter: 3        Sample Start Region (min): 0  Sample End Region (min): 35
Manual Baseline Start (min): 10     Manual Baseline End (min): 35
Marker Peak Width (sec): 5    Marker Min Peak Height: 500   Marker Baseline V to V?: N    Marker Baseline V to V pts: 3
Lower Marker Selection: First Peak > 500 RFU                Upper Marker Selection: Last Peak > 500 RFU
Ladder Size (bp): 1, 75, 200, 400, 600, 800, 1000, 1500, 3000, 6000, 10000, 15000, 48500, 200000
Quantification Using: Ladder          Final Concentration (ng/uL): 0.1250          Dilution Factor: 12.0
Min. RFU for Data Processing: 3
```

 Copyright 2015 Advanced Analytical Technologies, Inc.

Sample: 4021
Well Location: C5
Created: Thursday, July 21, 2016 2:23:11 PM

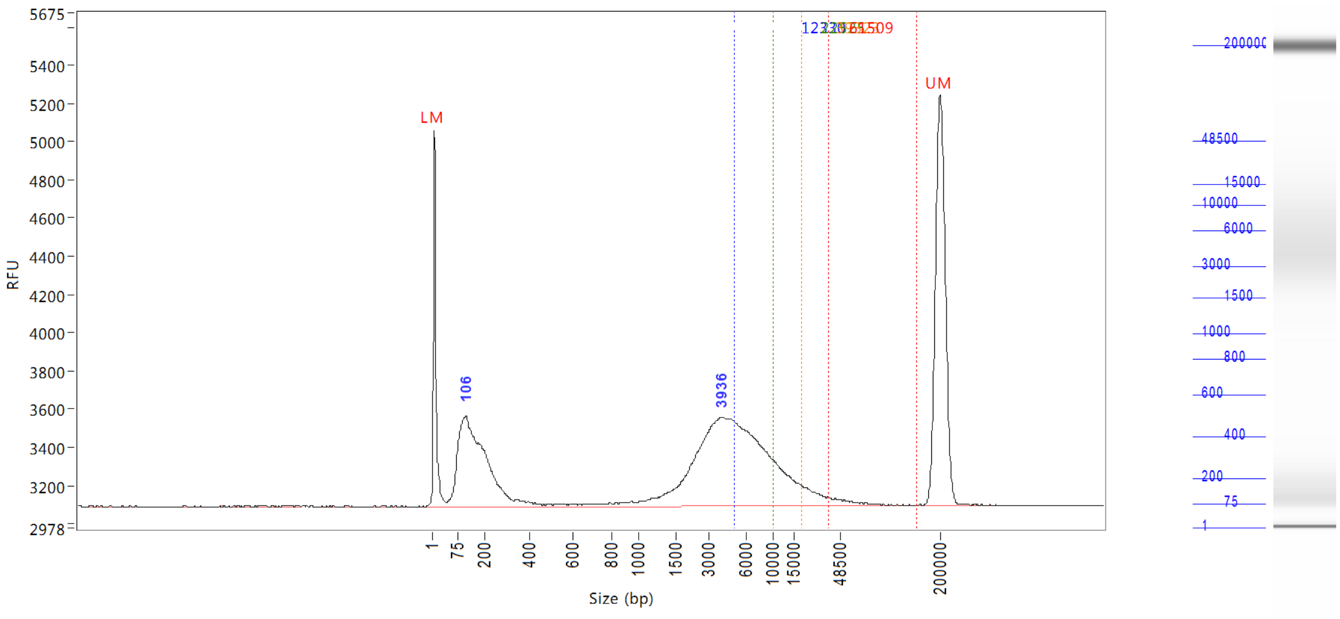

Peak	Size (bp)	Conc. (ng/uL)	From (bp)	To (bp)	Avg. Size (bp)	CV%	RFU	Corr. Peak Area
1	1 (LM)	0.0078	0	34	2	369.31	1967	15.781
2	106	0.2274	34	417	151	47.69	474	38.510
3	3936	0.3692	959	45979	6585	97.09	461	62.505
4	200000 (UM)	0.0140	174134	241308	200284	3.51	2145	28.546

TIC:	0.5966	ng/uL
TIM:	3.665	nmole/L
Total Conc.:	0.6136	ng/uL

Smear Analysis						
	40000 bp to 165000 bp	0.0075 ng/ul	1.2 %Total	0.000 nmole/L	61509 Avg. Size (b.p.)	36.03 %CV
	20000 bp to 165000 bp	0.0227 ng/ul	3.7 %Total	0.001 nmole/L	39323 Avg. Size (b.p.)	52.66 %CV
	10000 bp to 165000 bp	0.0625 ng/ul	10.2 %Total	0.005 nmole/L	22525 Avg. Size (b.p.)	79.29 %CV
	5000 bp to 165000 bp	0.1768 ng/ul	28.8 %Total	0.024 nmole/L	12330 Avg. Size (b.p.)	105.15 %CV

```
Sample Peak Width (sec): 10   Sample Min Peak Height: 50    Sample Baseline V to V?: Y    Sample Baseline V to V pts: 3
Sample Filter: Binomial       # of Pts for Filter: 3        Sample Start Region (min): 0  Sample End Region (min): 35
Manual Baseline Start (min): 10     Manual Baseline End (min): 35
Marker Peak Width (sec): 5    Marker Min Peak Height: 500   Marker Baseline V to V?: N    Marker Baseline V to V pts: 3
Lower Marker Selection: First Peak > 500 RFU                Upper Marker Selection: Last Peak > 500 RFU
Ladder Size (bp): 1, 75, 200, 400, 600, 800, 1000, 1500, 3000, 6000, 10000, 15000, 48500, 200000
Quantification Using: Ladder          Final Concentration (ng/uL): 0.1250          Dilution Factor: 12.0
Min. RFU for Data Processing: 3
```

 Copyright 2015 Advanced Analytical Technologies, Inc.

Sample: 1021
Well Location: C6
Created: Thursday, July 21, 2016 2:23:11 PM

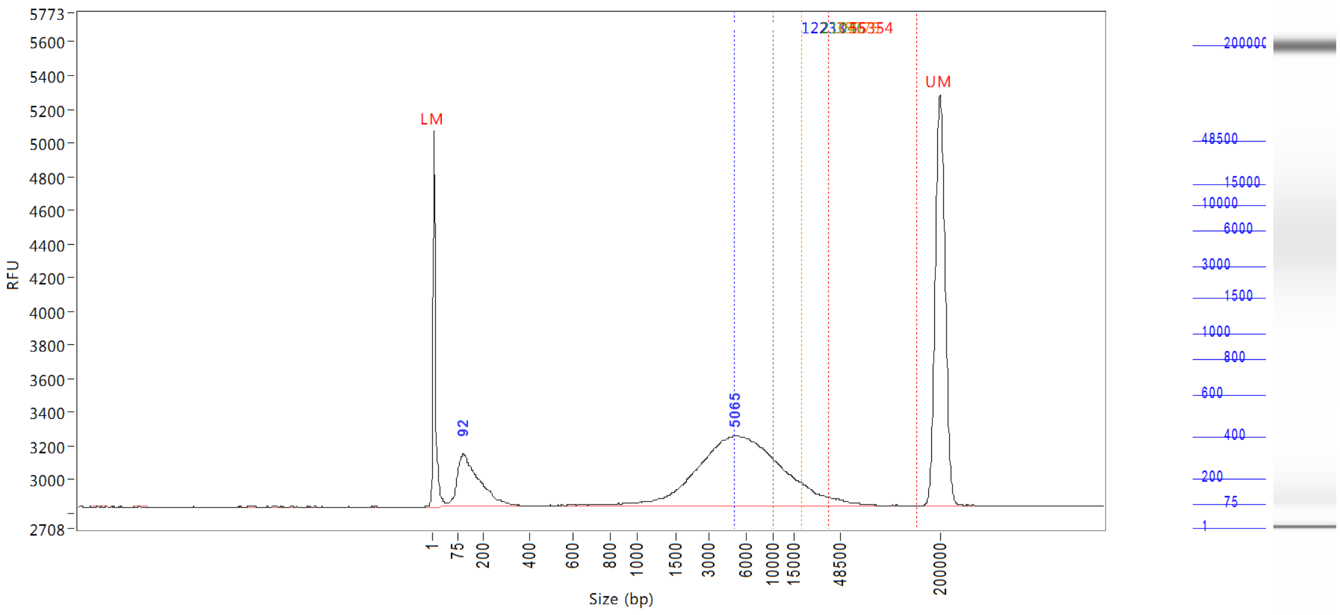

Peak	Size (bp)	Conc. (ng/uL)	From (bp)	To (bp)	Avg. Size (bp)	CV%	RFU	Corr. Peak Area
1	1 (LM)	0.0078	0	38	2	325.46	2229	17.884
2	92	0.1018	38	277	130	40.39	310	19.531
3	5065	0.3144	978	44898	7296	93.17	416	60.318
4	200000 (UM)	0.0141	173395	236882	200013	3.48	2438	32.377

TIC:	0.4161	ng/uL
TIM:	1.911	nmole/L
Total Conc.:	0.4315	ng/uL

Smear Analysis						
	40000 bp to 165000 bp	0.0066 ng/ul	1.5 %Total	0.000 nmole/L	55354 Avg. Size (b.p.)	29.21 %CV
	20000 bp to 165000 bp	0.0230 ng/ul	5.3 %Total	0.001 nmole/L	35975 Avg. Size (b.p.)	44.09 %CV
	10000 bp to 165000 bp	0.0657 ng/ul	15.2 %Total	0.005 nmole/L	21046 Avg. Size (b.p.)	68.96 %CV
	5000 bp to 165000 bp	0.1744 ng/ul	40.4 %Total	0.023 nmole/L	12233 Avg. Size (b.p.)	92.01 %CV

```
Sample Peak Width (sec): 10   Sample Min Peak Height: 50    Sample Baseline V to V?: Y    Sample Baseline V to V pts: 3
Sample Filter: Binomial       # of Pts for Filter: 3        Sample Start Region (min): 0  Sample End Region (min): 35
Manual Baseline Start (min): 10     Manual Baseline End (min): 35
Marker Peak Width (sec): 5    Marker Min Peak Height: 500   Marker Baseline V to V?: N    Marker Baseline V to V pts: 3
Lower Marker Selection: First Peak > 500 RFU                Upper Marker Selection: Last Peak > 500 RFU
Ladder Size (bp): 1, 75, 200, 400, 600, 800, 1000, 1500, 3000, 6000, 10000, 15000, 48500, 200000
Quantification Using: Ladder          Final Concentration (ng/uL): 0.1250          Dilution Factor: 12.0
Min. RFU for Data Processing: 3
```

 Copyright 2015 Advanced Analytical Technologies, Inc.

Sample: 1022.1
Well Location: C7
Created: Thursday, July 21, 2016 2:23:11 PM

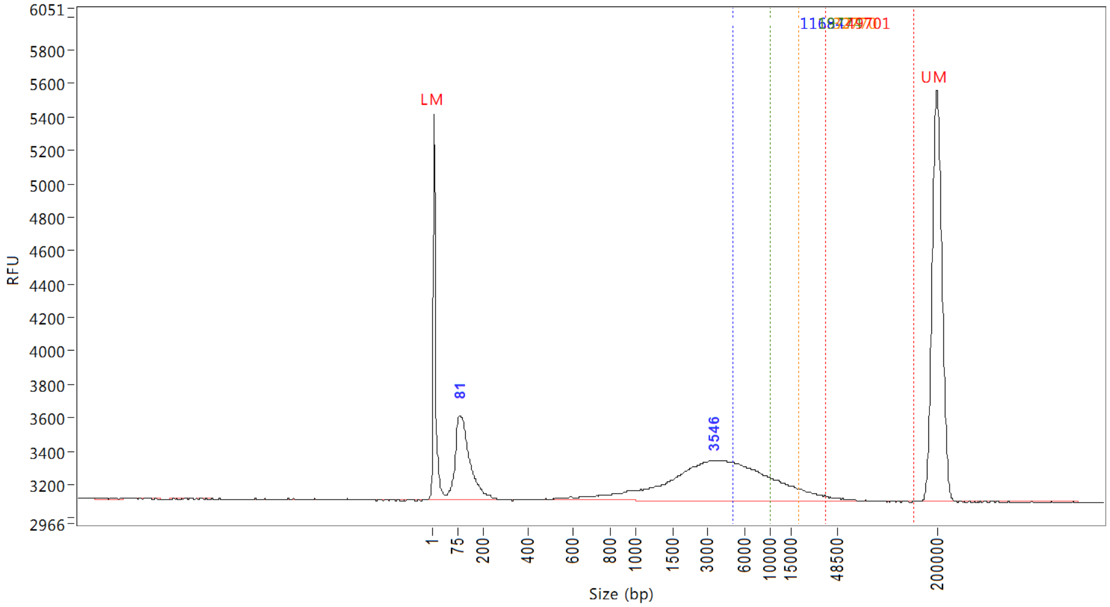

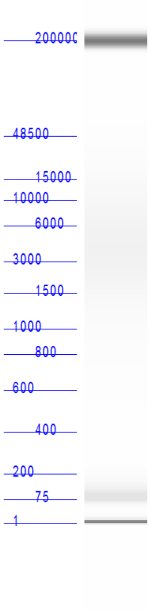

Peak	Size (bp)	Conc. (ng/uL)	From (bp)	To (bp)	Avg. Size (bp)	CV%	RFU	Corr. Peak Area
1	1 (LM)	0.0078	0	37	2	296.13	2303	18.580
2	81	0.1085	37	324	100	36.63	496	21.627
3	3546	0.2206	700	64020	5846	120.60	239	43.976
4	200000 (UM)	0.0135	177090	231718	200331	3.39	2456	32.348

TIC:	0.3291	ng/uL
TIM:	2.309	nmole/L
Total Conc.:	0.3343	ng/uL

Smear Analysis						
	40000 bp to 165000 bp	0.0023 ng/ul	0.7 %Total	0.000 nmole/L	49701 Avg. Size (b.p.)	18.33 %CV
	20000 bp to 165000 bp	0.0105 ng/ul	3.1 %Total	0.001 nmole/L	32770 Avg. Size (b.p.)	33.26 %CV
	10000 bp to 165000 bp	0.0310 ng/ul	9.3 %Total	0.003 nmole/L	19774 Avg. Size (b.p.)	57.89 %CV
	5000 bp to 165000 bp	0.0827 ng/ul	24.7 %Total	0.012 nmole/L	11684 Avg. Size (b.p.)	80.60 %CV

```
Sample Peak Width (sec): 10   Sample Min Peak Height: 50    Sample Baseline V to V?: Y    Sample Baseline V to V pts: 3
Sample Filter: Binomial       # of Pts for Filter: 3        Sample Start Region (min): 0  Sample End Region (min): 35
Manual Baseline Start (min): 10     Manual Baseline End (min): 35
Marker Peak Width (sec): 5    Marker Min Peak Height: 500   Marker Baseline V to V?: N    Marker Baseline V to V pts: 3
Lower Marker Selection: First Peak > 500 RFU                Upper Marker Selection: Last Peak > 500 RFU
Ladder Size (bp): 1, 75, 200, 400, 600, 800, 1000, 1500, 3000, 6000, 10000, 15000, 48500, 200000
Quantification Using: Ladder          Final Concentration (ng/uL): 0.1250          Dilution Factor: 12.0
Min. RFU for Data Processing: 3
```

 Copyright 2015 Advanced Analytical Technologies, Inc.

Sample: 4023.1
Well Location: C8
Created: Thursday, July 21, 2016 2:23:11 PM

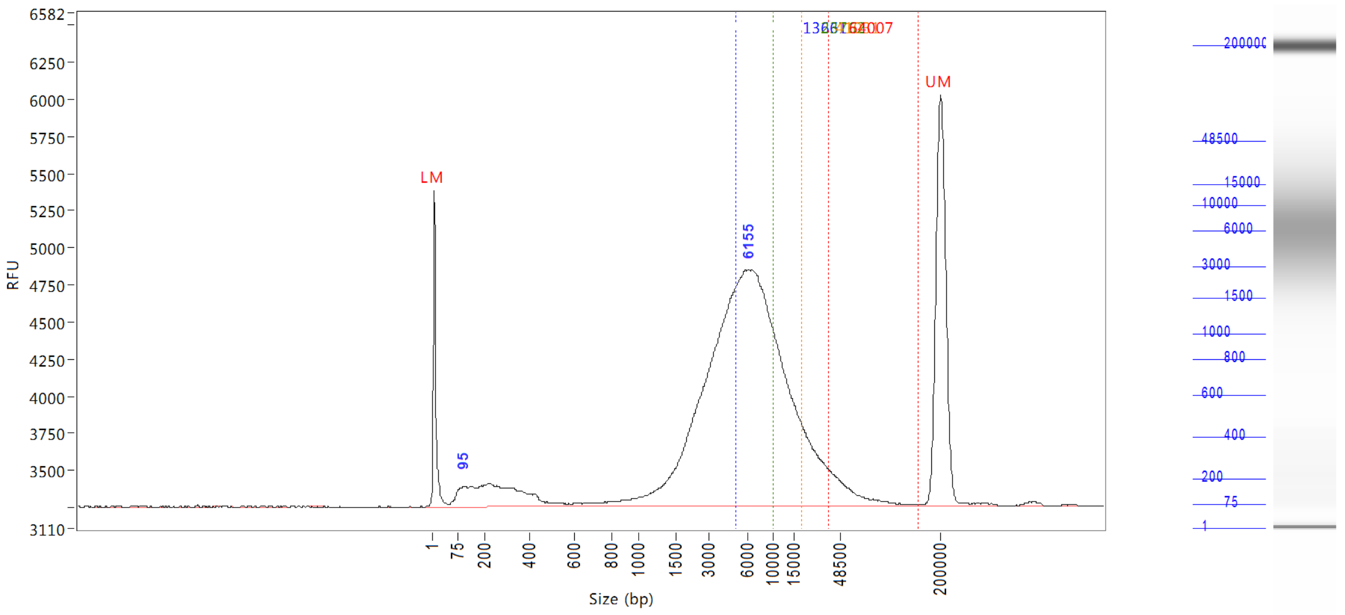

Peak	Size (bp)	Conc. (ng/uL)	From (bp)	To (bp)	Avg. Size (bp)	CV%	RFU	Corr. Peak Area
1	1 (LM)	0.0078	0	41	2	431.79	2125	16.841
2	95	0.0417	41	160	105	29.73	134	7.534
3	6155	1.2559	797	92103	8984	115.65	1593	226.939
4	200000 (UM)	0.0171	168222	244258	200454	3.74	2762	37.147

TIC:	1.2976	ng/uL
TIM:	1.059	nmole/L
Total Conc.:	1.4129	ng/uL

Smear Analysis						
	40000 bp to 165000 bp	0.0415 ng/ul	2.9 %Total	0.001 nmole/L	64007 Avg. Size (b.p.)	41.04 %CV
	20000 bp to 165000 bp	0.1179 ng/ul	8.3 %Total	0.005 nmole/L	41031 Avg. Size (b.p.)	57.23 %CV
	10000 bp to 165000 bp	0.3073 ng/ul	21.8 %Total	0.021 nmole/L	23762 Avg. Size (b.p.)	84.20 %CV
	5000 bp to 165000 bp	0.7699 ng/ul	54.5 %Total	0.093 nmole/L	13661 Avg. Size (b.p.)	110.30 %CV

```
Sample Peak Width (sec): 10   Sample Min Peak Height: 50    Sample Baseline V to V?: Y    Sample Baseline V to V pts: 3
Sample Filter: Binomial       # of Pts for Filter: 3        Sample Start Region (min): 0  Sample End Region (min): 35
Manual Baseline Start (min): 10     Manual Baseline End (min): 35
Marker Peak Width (sec): 5    Marker Min Peak Height: 500   Marker Baseline V to V?: N    Marker Baseline V to V pts: 3
Lower Marker Selection: First Peak > 500 RFU                Upper Marker Selection: Last Peak > 500 RFU
Ladder Size (bp): 1, 75, 200, 400, 600, 800, 1000, 1500, 3000, 6000, 10000, 15000, 48500, 200000
Quantification Using: Ladder         Final Concentration (ng/uL): 0.1250         Dilution Factor: 12.0
Min. RFU for Data Processing: 3
```

 Copyright 2015 Advanced Analytical Technologies, Inc.

Sample: 1014.2
Well Location: C9
Created: Thursday, July 21, 2016 2:23:11 PM

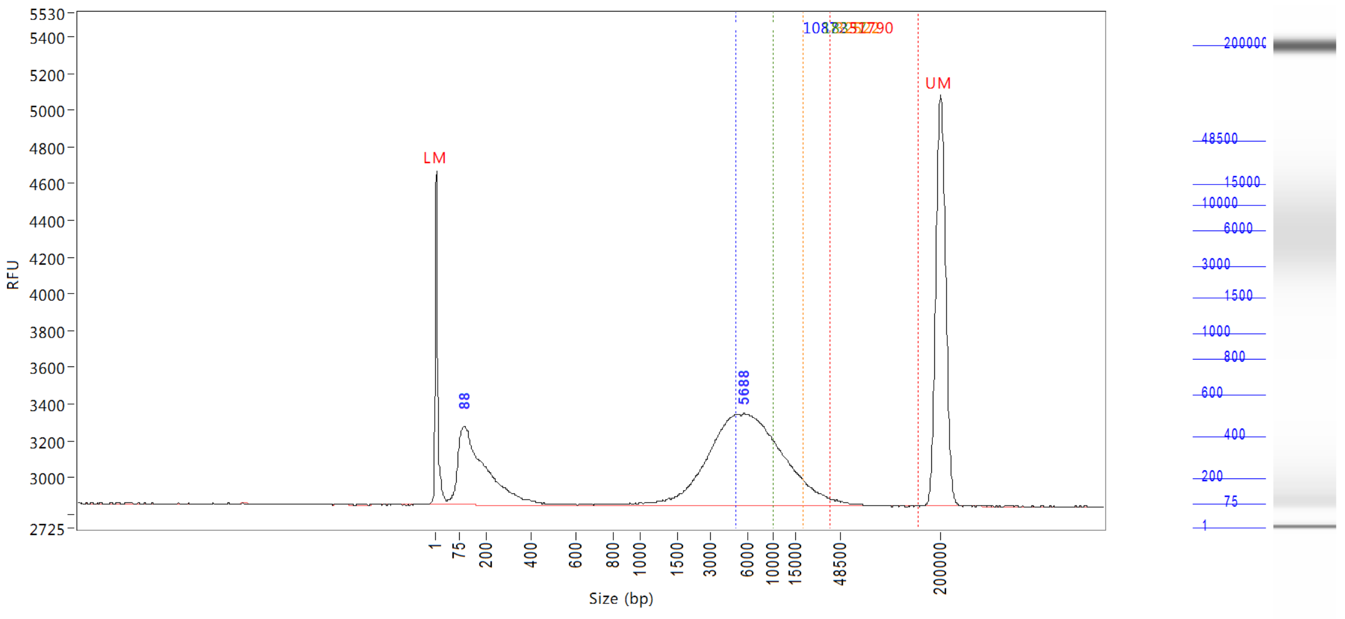

Peak	Size (bp)	Conc. (ng/uL)	From (bp)	To (bp)	Avg. Size (bp)	CV%	RFU	Corr. Peak Area
1	1 (LM)	0.0078	0	31	1	729.88	1817	14.385
2	88	0.2135	31	383	149	51.15	423	32.954
3	5688	0.4131	1040	44178	7518	82.02	498	63.760
4	200000 (UM)	0.0160	168222	233931	200094	3.45	2235	29.574

TIC:	0.6266	ng/uL
TIM:	4.116	nmole/L
Total Conc.:	0.6362	ng/uL

Smear Analysis						
	40000 bp to 165000 bp	0.0047 ng/ul	0.7 %Total	0.000 nmole/L	51790 Avg. Size (b.p.)	25.20 %CV
	20000 bp to 165000 bp	0.0242 ng/ul	3.8 %Total	0.001 nmole/L	32522 Avg. Size (b.p.)	37.63 %CV
	10000 bp to 165000 bp	0.0876 ng/ul	13.8 %Total	0.008 nmole/L	18337 Avg. Size (b.p.)	60.10 %CV
	5000 bp to 165000 bp	0.2513 ng/ul	39.5 %Total	0.038 nmole/L	10872 Avg. Size (b.p.)	78.09 %CV

```
Sample Peak Width (sec): 10   Sample Min Peak Height: 50    Sample Baseline V to V?: Y    Sample Baseline V to V pts: 3
Sample Filter: Binomial       # of Pts for Filter: 3        Sample Start Region (min): 0  Sample End Region (min): 35
Manual Baseline Start (min): 10     Manual Baseline End (min): 35
Marker Peak Width (sec): 5    Marker Min Peak Height: 500   Marker Baseline V to V?: N    Marker Baseline V to V pts: 3
Lower Marker Selection: First Peak > 500 RFU                Upper Marker Selection: Last Peak > 500 RFU
Ladder Size (bp): 1, 75, 200, 400, 600, 800, 1000, 1500, 3000, 6000, 10000, 15000, 48500, 200000
Quantification Using: Ladder          Final Concentration (ng/uL): 0.1250          Dilution Factor: 12.0
Min. RFU for Data Processing: 3
```


Copyright 2015 Advanced Analytical Technologies, Inc.

Sample: 6037.3
Well Location: C10
Created: Thursday, July 21, 2016 2:23:11 PM

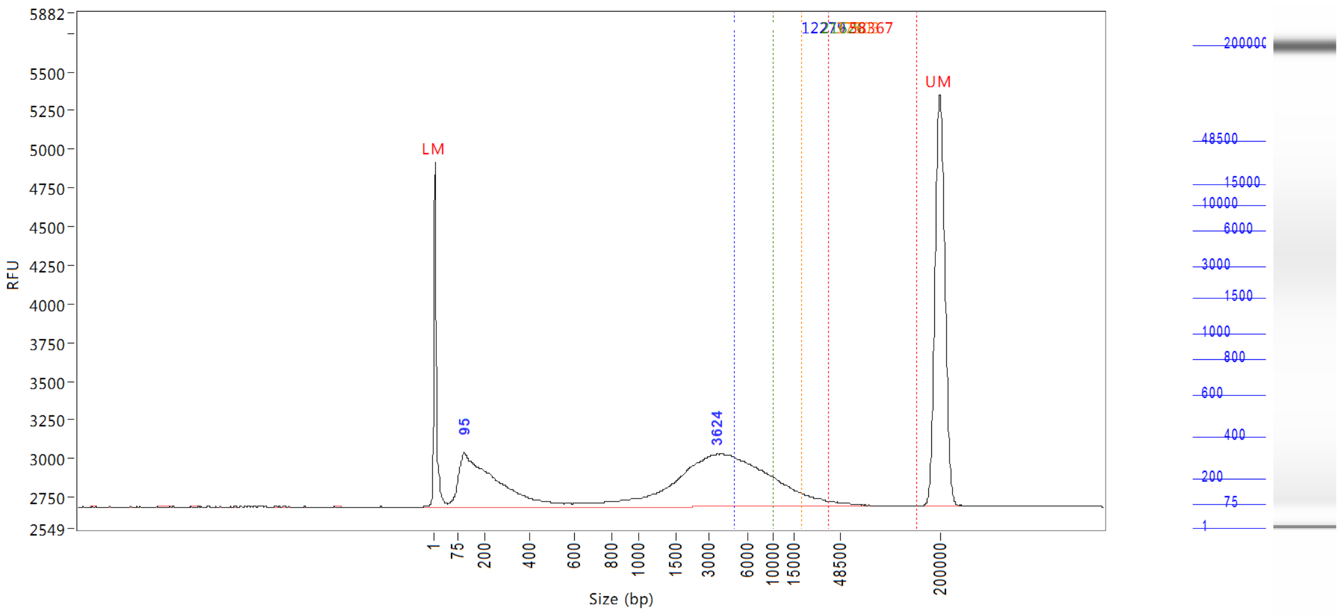

Peak	Size (bp)	Conc. (ng/uL)	From (bp)	To (bp)	Avg. Size (bp)	CV%	RFU	Corr. Peak Area
1	1 (LM)	0.0078	0	38	2	397.51	2226	17.713
2	95	0.1638	38	313	162	43.27	346	31.127
3	3624	0.3034	664	42016	5561	105.21	338	57.654
4	200000 (UM)	0.0156	169700	252373	199735	3.55	2656	35.483

TIC:	0.4672	ng/uL
TIM:	2.977	nmole/L
Total Conc.:	0.5049	ng/uL

Smear Analysis						
	40000 bp to 165000 bp	0.0047 ng/ul	0.9 %Total	0.000 nmole/L	58367 Avg. Size (b.p.)	36.09 %CV
	20000 bp to 165000 bp	0.0154 ng/ul	3.0 %Total	0.001 nmole/L	37503 Avg. Size (b.p.)	49.80 %CV
	10000 bp to 165000 bp	0.0431 ng/ul	8.5 %Total	0.003 nmole/L	21626 Avg. Size (b.p.)	75.02 %CV
	5000 bp to 165000 bp	0.1183 ng/ul	23.4 %Total	0.016 nmole/L	12279 Avg. Size (b.p.)	98.93 %CV

```
Sample Peak Width (sec): 10   Sample Min Peak Height: 50    Sample Baseline V to V?: Y    Sample Baseline V to V pts: 3
Sample Filter: Binomial       # of Pts for Filter: 3        Sample Start Region (min): 0  Sample End Region (min): 35
Manual Baseline Start (min): 10     Manual Baseline End (min): 35
Marker Peak Width (sec): 5    Marker Min Peak Height: 500   Marker Baseline V to V?: N    Marker Baseline V to V pts: 3
Lower Marker Selection: First Peak > 500 RFU                Upper Marker Selection: Last Peak > 500 RFU
Ladder Size (bp): 1, 75, 200, 400, 600, 800, 1000, 1500, 3000, 6000, 10000, 15000, 48500, 200000
Quantification Using: Ladder          Final Concentration (ng/uL): 0.1250          Dilution Factor: 12.0
Min. RFU for Data Processing: 3
```


Copyright 2015 Advanced Analytical Technologies, Inc.

Sample: 4024.1
Well Location: C11
Created: Thursday, July 21, 2016 2:23:11 PM

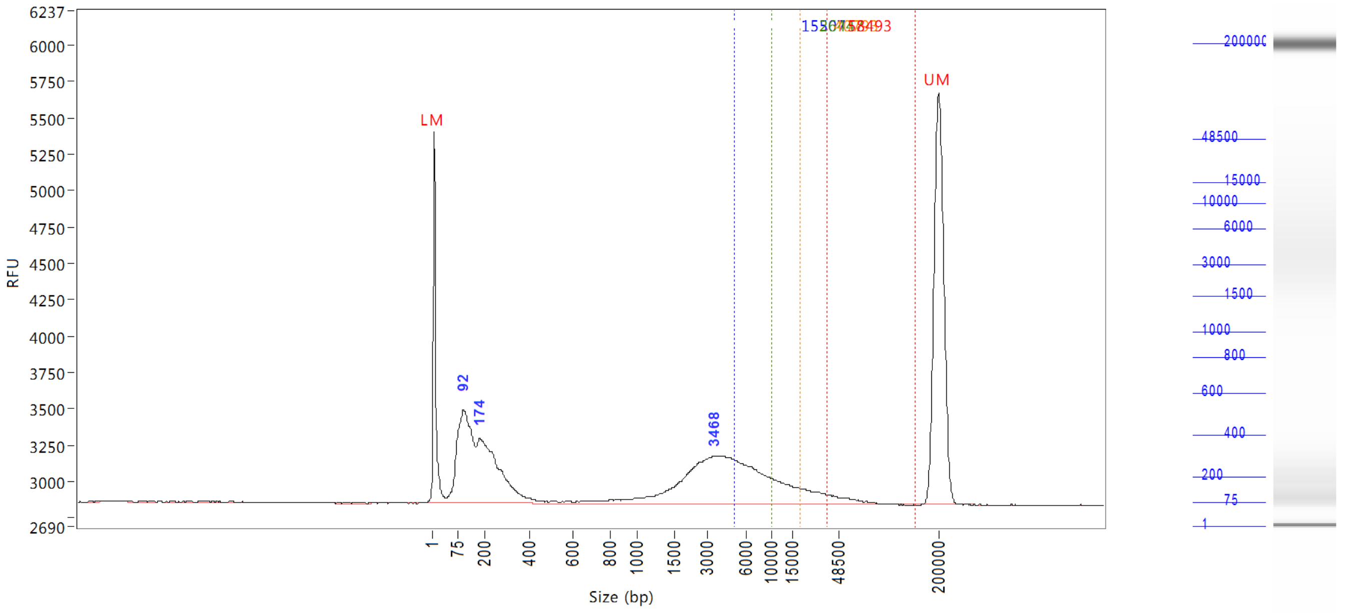

Peak	Size (bp)	Conc. (ng/uL)	From (bp)	To (bp)	Avg. Size (bp)	CV%	RFU	Corr. Peak Area
1	1 (LM)	0.0078	0	33	1	590.65	2548	20.535
2	92	0.1346	33	156	100	28.99	643	29.659
3	174	0.1299	156	439	232	25.16	442	28.617
4	3468	0.2433	871	115752	8451	145.46	329	53.596
5	200000 (UM)	0.0142	166744	252373	200258	3.43	2827	37.419

TIC:	0.5078	ng/uL
TIM:	3.733	nmole/L
Total Conc.:	0.5146	ng/uL

Smear Analysis						
	40000 bp to 165000 bp	0.0092 ng/ul	1.8 %Total	0.000 nmole/L	58493 Avg. Size (b.p.)	28.95 %CV
	20000 bp to 165000 bp	0.0231 ng/ul	4.5 %Total	0.001 nmole/L	40793 Avg. Size (b.p.)	45.29 %CV
	10000 bp to 165000 bp	0.0482 ng/ul	9.4 %Total	0.003 nmole/L	26447 Avg. Size (b.p.)	71.41 %CV
	5000 bp to 165000 bp	0.1087 ng/ul	21.1 %Total	0.012 nmole/L	15507 Avg. Size (b.p.)	102.53 %CV

```
Sample Peak Width (sec): 10   Sample Min Peak Height: 50    Sample Baseline V to V?: Y    Sample Baseline V to V pts: 3
Sample Filter: Binomial       # of Pts for Filter: 3        Sample Start Region (min): 0  Sample End Region (min): 35
Manual Baseline Start (min): 10     Manual Baseline End (min): 35
Marker Peak Width (sec): 5    Marker Min Peak Height: 500   Marker Baseline V to V?: N    Marker Baseline V to V pts: 3
Lower Marker Selection: First Peak > 500 RFU                Upper Marker Selection: Last Peak > 500 RFU
Ladder Size (bp): 1, 75, 200, 400, 600, 800, 1000, 1500, 3000, 6000, 10000, 15000, 48500, 200000
Quantification Using: Ladder          Final Concentration (ng/uL): 0.1250          Dilution Factor: 12.0
Min. RFU for Data Processing: 3
```

 Copyright 2015 Advanced Analytical Technologies, Inc.

Sample: HS LF Ladder
Well Location: C12
Created: Thursday, July 21, 2016 2:23:11 PM

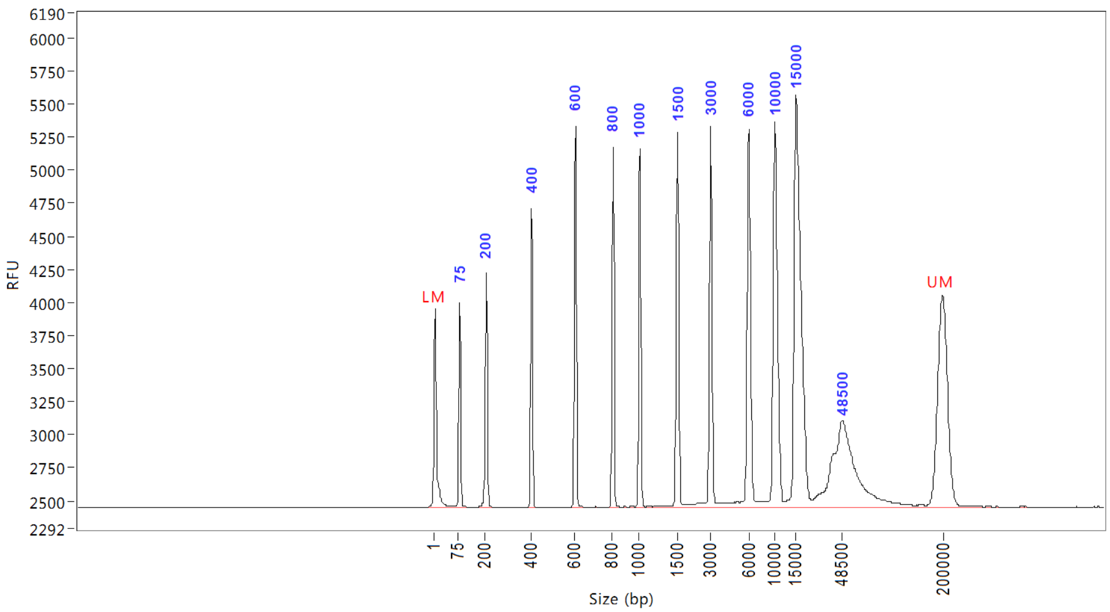

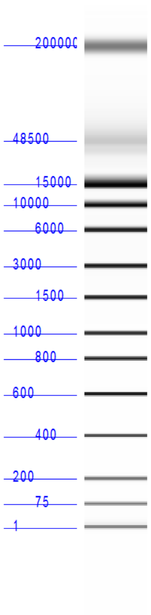

Peak	Size (bp)	Conc. (ng/uL)	From (bp)	To (bp)	Avg. Size (bp)	CV%	RFU	Corr. Peak Area
1	1 (LM)	0.0078	0	65	2	344.81	1495	12.307
2	75	0.0671	65	144	73	5.05	1549	8.854
3	200	0.0809	144	275	197	2.53	1766	10.687
4	400	0.0827	383	428	398	0.88	2262	10.916
5	600	0.1058	566	750	598	0.93	2878	13.971
6	800	0.0983	763	867	797	0.67	2717	12.981
7	1000	0.0993	867	1159	996	1.09	2709	13.113
8	1500	0.1065	1375	1702	1496	1.70	2828	14.066
9	3000	0.1233	2060	4286	2988	7.08	2872	16.281
10	6000	0.1273	5260	7001	5981	3.09	2859	16.806
11	10000	0.1598	8616	12210	10091	4.38	2912	21.095
12	15000	0.2596	12210	26528	16413	13.85	3111	34.284
13	48500	0.1734	26528	126098	56081	33.81	655	22.899
14	200000 (UM)	0.0136	174134	250898	199762	3.69	1601	21.482

TIC:	1.4840	ng/uL
TIM:	3.408	nmole/L
Total Conc.:	1.5000	ng/uL

```
Sample Peak Width (sec): 10   Sample Min Peak Height: 200   Sample Baseline V to V?: Y    Sample Baseline V to V pts: 3
Sample Filter: Binomial       # of Pts for Filter: 3        Sample Start Region (min): 0  Sample End Region (min): 35
Manual Baseline Start (min): 10     Manual Baseline End (min): 35
Marker Peak Width (sec): 5    Marker Min Peak Height: 500   Marker Baseline V to V?: N    Marker Baseline V to V pts: 3
Lower Marker Selection: First Peak > 500 RFU                Upper Marker Selection: Last Peak > 500 RFU
Ladder Size (bp): 1, 75, 200, 400, 600, 800, 1000, 1500, 3000, 6000, 10000, 15000, 48500, 200000
Quantification Using: Ladder         Final Concentration (ng/uL): 0.1250         Dilution Factor: 12.0
Min. RFU for Data Processing: 3
```


Copyright 2015 Advanced Analytical Technologies, Inc.

Sample: HS LF Ladder
Well Location: C12
Created: Thursday, July 21, 2016 2:23:11 PM
Fit Type: Point to Point

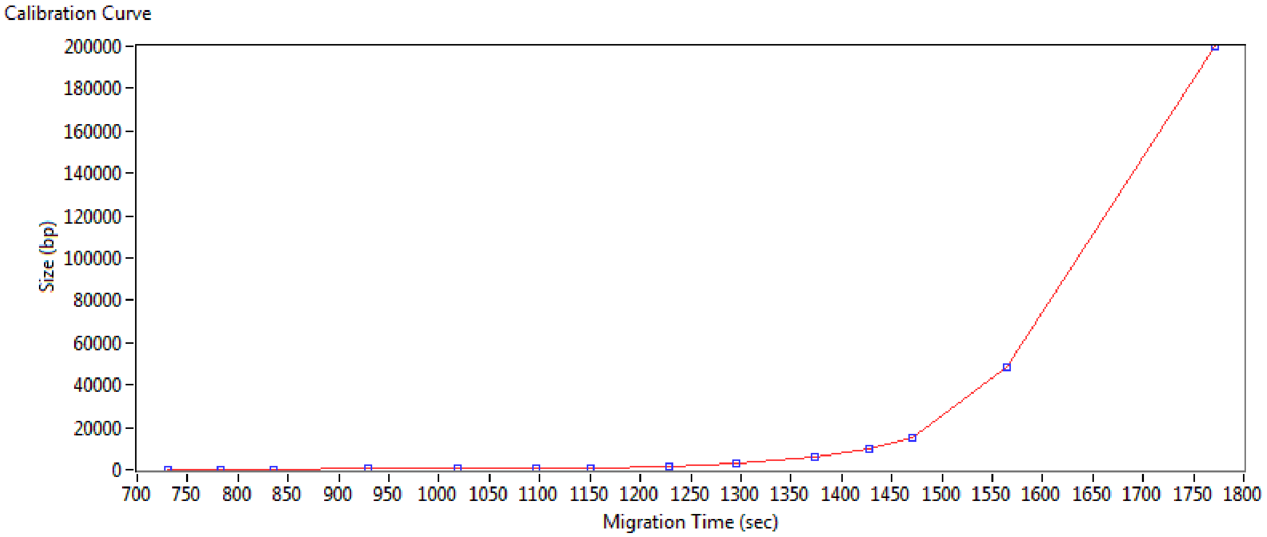

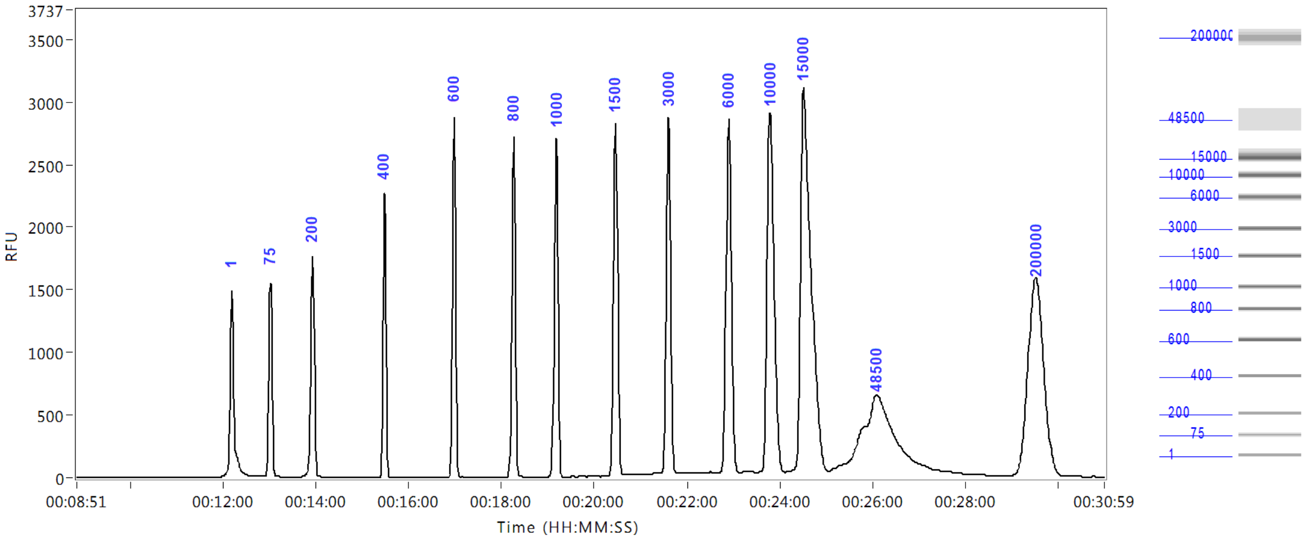

 Copyright 2015 Advanced Analytical Technologies, Inc.
