# Supplementary material for: Longitudinal linked-read sequencing reveals ecological and evolutionary responses of a human gut microbiome during antibiotic treatment
Source: Genome Res. 2021 Aug;31(8):1433–46. doi: 10.1101/gr.265058.120 (PMC8327913; doi:10.1101/gr.265058.120)
Supplement: Supplemental Material [file supp_gr.265058.120_Supplemental_Data_S6.pdf]

Sample: 1025
Well Location: E1
Created: Thursday, July 21, 2016 3:14:29 PM

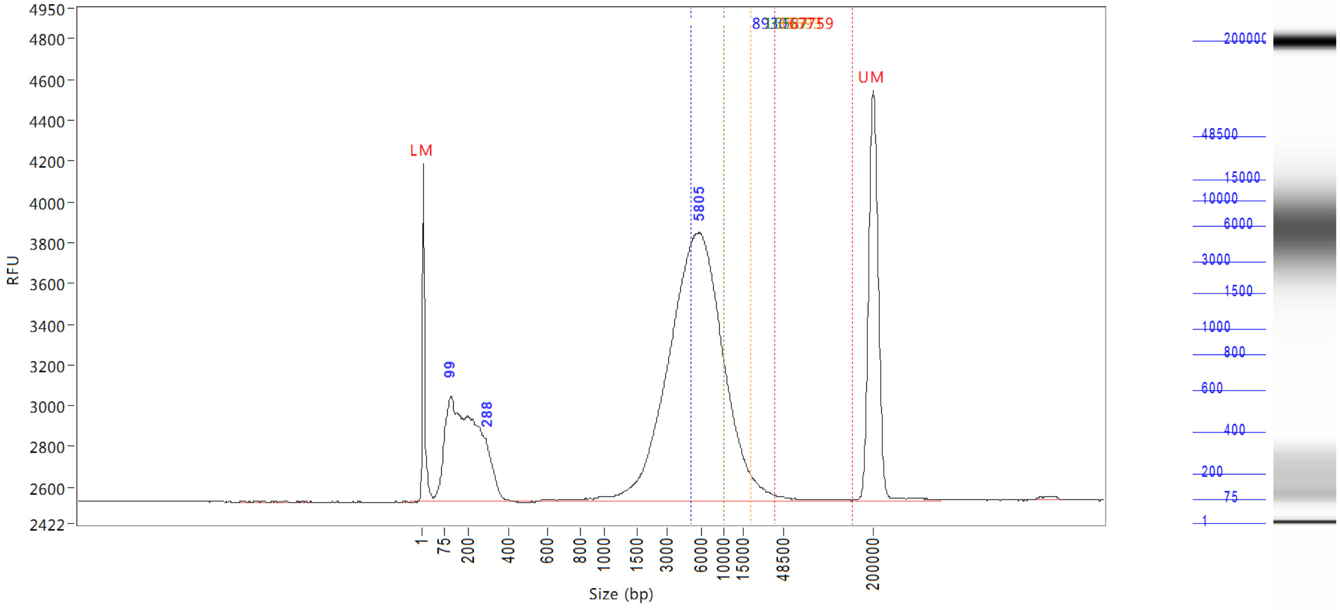

Peak	Size (bp)	Conc. (ng/uL)	From (bp)	To (bp)	Avg. Size (bp)	CV%	RFU	Corr. Peak Area
1	1 (LM)	0.0078	0	34	1	509.31	1658	12.919
2	99	0.3397	34	288	163	41.00	513	47.085
3	288	0.0402	288	468	313	6.67	293	5.574
4	5805	0.9667	1034	64020	6401	73.65	1318	133.998
5	200000 (UM)	0.0167	169700	256799	200203	4.05	2008	27.733

TIC:	1.3466	ng/uL
TIM:	6.116	nmole/L
Total Conc.:	1.3533	ng/uL

Smear Analysis						
	40000 bp to 165000 bp	0.0044 ng/ul	0.3 %Total	0.000 nmole/L	67759 Avg. Size (b.p.)	49.73 %CV
	20000 bp to 165000 bp	0.0216 ng/ul	1.6 %Total	0.001 nmole/L	35693 Avg. Size (b.p.)	64.28 %CV
	10000 bp to 165000 bp	0.1208 ng/ul	8.9 %Total	0.012 nmole/L	16587 Avg. Size (b.p.)	79.67 %CV
	5000 bp to 165000 bp	0.5551 ng/ul	41.0 %Total	0.102 nmole/L	8930 Avg. Size (b.p.)	82.78 %CV

```
Sample Peak Width (sec): 10   Sample Min Peak Height: 50    Sample Baseline V to V?: Y    Sample Baseline V to V pts: 3
Sample Filter: Binomial       # of Pts for Filter: 3        Sample Start Region (min): 0  Sample End Region (min): 35
Manual Baseline Start (min): 10     Manual Baseline End (min): 35
Marker Peak Width (sec): 5    Marker Min Peak Height: 500   Marker Baseline V to V?: N    Marker Baseline V to V pts: 3
Lower Marker Selection: First Peak > 500 RFU                Upper Marker Selection: Last Peak > 500 RFU
Ladder Size (bp): 1, 75, 200, 400, 600, 800, 1000, 1500, 3000, 6000, 10000, 15000, 48500, 200000
Quantification Using: Ladder          Final Concentration (ng/uL): 0.1250          Dilution Factor: 12.0
Min. RFU for Data Processing: 3
```

Sample: 1023
Well Location: E2
Created: Thursday, July 21, 2016 3:14:29 PM

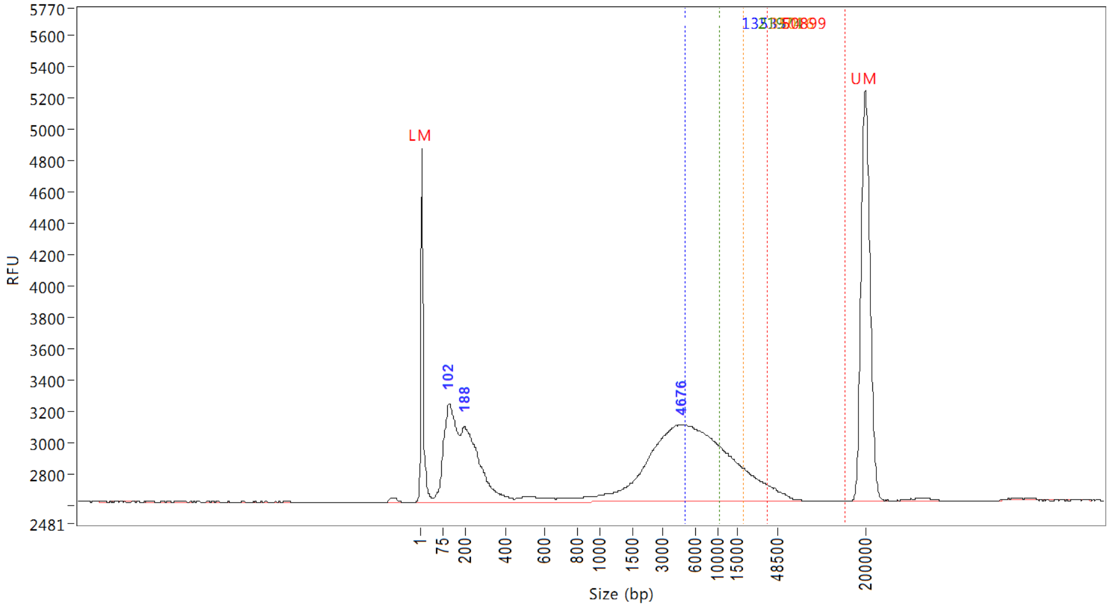

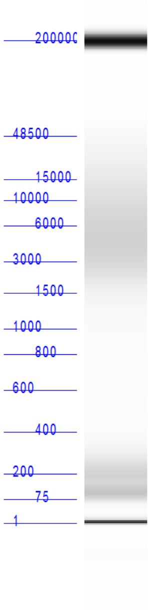

Peak	Size (bp)	Conc. (ng/uL)	From (bp)	To (bp)	Avg. Size (bp)	CV%	RFU	Corr. Peak Area
1	1 (LM)	0.0078	0	37	2	451.52	2259	17.890
2	102	0.1352	37	160	108	27.00	629	25.959
3	188	0.1441	160	441	234	24.49	482	27.665
4	4676	0.4076	729	96537	8432	117.92	489	78.242
5	200000 (UM)	0.0150	170439	246472	200086	3.62	2615	34.600

TIC:	0.6870	ng/uL
TIM:	3.585	nmole/L
Total Conc.:	0.6975	ng/uL

Smear Analysis						
	40000 bp to 165000 bp	0.0109 ng/ul	1.6 %Total	0.000 nmole/L	50899 Avg. Size (b.p.)	20.33 %CV
	20000 bp to 165000 bp	0.0380 ng/ul	5.4 %Total	0.002 nmole/L	35016 Avg. Size (b.p.)	35.51 %CV
	10000 bp to 165000 bp	0.0944 ng/ul	13.5 %Total	0.007 nmole/L	21974 Avg. Size (b.p.)	61.12 %CV
	5000 bp to 165000 bp	0.2141 ng/ul	30.7 %Total	0.026 nmole/L	13533 Avg. Size (b.p.)	85.94 %CV

```
Sample Peak Width (sec): 10   Sample Min Peak Height: 50    Sample Baseline V to V?: Y    Sample Baseline V to V pts: 3
Sample Filter: Binomial       # of Pts for Filter: 3        Sample Start Region (min): 0  Sample End Region (min): 35
Manual Baseline Start (min): 10     Manual Baseline End (min): 35
Marker Peak Width (sec): 5    Marker Min Peak Height: 500   Marker Baseline V to V?: N    Marker Baseline V to V pts: 3
Lower Marker Selection: First Peak > 500 RFU                Upper Marker Selection: Last Peak > 500 RFU
Ladder Size (bp): 1, 75, 200, 400, 600, 800, 1000, 1500, 3000, 6000, 10000, 15000, 48500, 200000
Quantification Using: Ladder          Final Concentration (ng/uL): 0.1250          Dilution Factor: 12.0
Min. RFU for Data Processing: 3
```

 Copyright 2015 Advanced Analytical Technologies, Inc.

Sample: 4026.2
Well Location: E3
Created: Thursday, July 21, 2016 3:14:29 PM

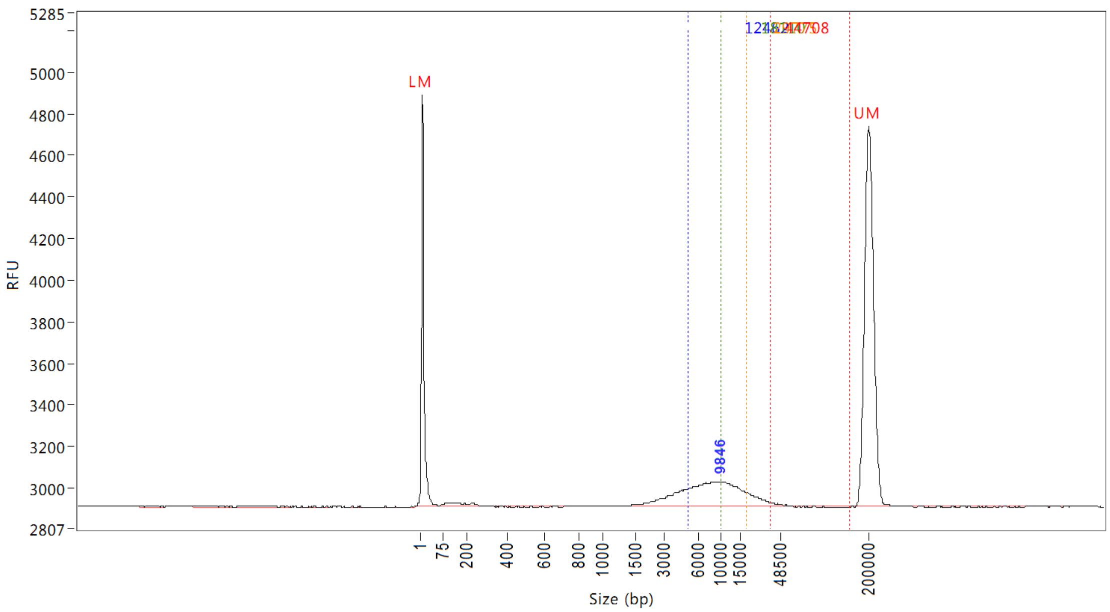

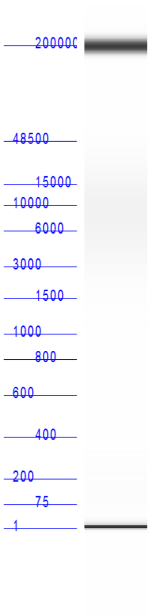

Peak	Size (bp)	Conc. (ng/uL)	From (bp)	To (bp)	Avg. Size (bp)	CV%	RFU	Corr. Peak Area
1	1 (LM)	0.0078	0	53	2	376.88	1975	15.547
2	9846	0.0842	1185	47059	10149	77.71	115	14.039
3	200000 (UM)	0.0120	174873	268602	199999	3.64	1824	24.113

TIC:	0.0842	ng/uL
TIM:	0.014	nmole/L
Total Conc.:	0.0918	ng/uL

Smear Analysis						
	40000 bp to 165000 bp	0.0011 ng/ul	1.2 %Total	0.000 nmole/L	44708 Avg. Size (b.p.)	8.67 %CV
	20000 bp to 165000 bp	0.0095 ng/ul	10.3 %Total	0.001 nmole/L	29775 Avg. Size (b.p.)	25.76 %CV
	10000 bp to 165000 bp	0.0312 ng/ul	34.1 %Total	0.003 nmole/L	18110 Avg. Size (b.p.)	49.22 %CV
	5000 bp to 165000 bp	0.0639 ng/ul	69.7 %Total	0.008 nmole/L	12462 Avg. Size (b.p.)	66.49 %CV

```
Sample Peak Width (sec): 10   Sample Min Peak Height: 50    Sample Baseline V to V?: Y    Sample Baseline V to V pts: 3
Sample Filter: Binomial       # of Pts for Filter: 3        Sample Start Region (min): 0  Sample End Region (min): 35
Manual Baseline Start (min): 10     Manual Baseline End (min): 35
Marker Peak Width (sec): 5    Marker Min Peak Height: 500   Marker Baseline V to V?: N    Marker Baseline V to V pts: 3
Lower Marker Selection: First Peak > 500 RFU                Upper Marker Selection: Last Peak > 500 RFU
Ladder Size (bp): 1, 75, 200, 400, 600, 800, 1000, 1500, 3000, 6000, 10000, 15000, 48500, 200000
Quantification Using: Ladder          Final Concentration (ng/uL): 0.1250          Dilution Factor: 12.0
Min. RFU for Data Processing: 3
```

 Copyright 2015 Advanced Analytical Technologies, Inc.

Sample: 1022
Well Location: E4
Created: Thursday, July 21, 2016 3:14:29 PM

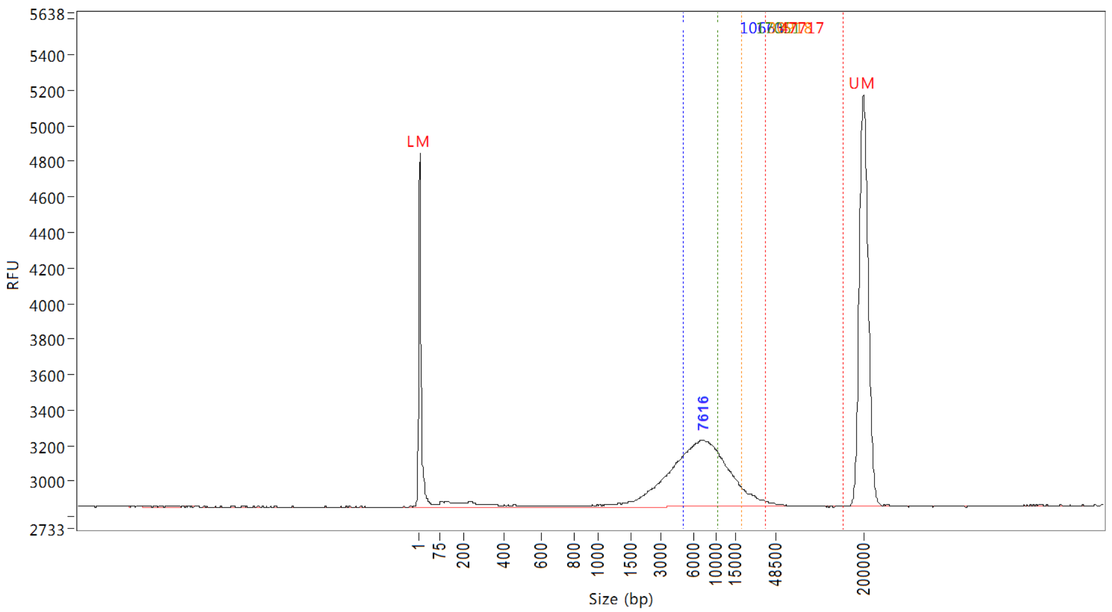

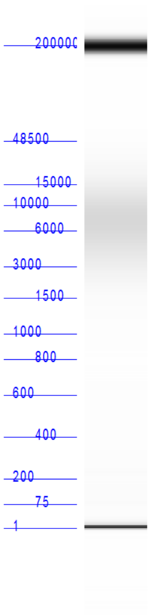

Peak	Size (bp)	Conc. (ng/uL)	From (bp)	To (bp)	Avg. Size (bp)	CV%	RFU	Corr. Peak Area
1	1 (LM)	0.0078	0	55	2	377.16	1988	15.887
2	7616	0.2408	1185	58847	8377	80.48	372	41.036
3	200000 (UM)	0.0150	174873	231718	200080	3.63	2312	30.710

TIC:	0.2408	ng/uL
TIM:	0.052	nmole/L
Total Conc.:	0.2649	ng/uL

Smear Analysis						
	40000 bp to 165000 bp	0.0022 ng/ul	0.8 %Total	0.000 nmole/L	47717 Avg. Size (b.p.)	17.68 %CV
	20000 bp to 165000 bp	0.0140 ng/ul	5.3 %Total	0.001 nmole/L	30918 Avg. Size (b.p.)	30.51 %CV
	10000 bp to 165000 bp	0.0603 ng/ul	22.8 %Total	0.006 nmole/L	17051 Avg. Size (b.p.)	53.56 %CV
	5000 bp to 165000 bp	0.1673 ng/ul	63.2 %Total	0.026 nmole/L	10665 Avg. Size (b.p.)	68.50 %CV

```
Sample Peak Width (sec): 10   Sample Min Peak Height: 50    Sample Baseline V to V?: Y    Sample Baseline V to V pts: 3
Sample Filter: Binomial       # of Pts for Filter: 3        Sample Start Region (min): 0  Sample End Region (min): 35
Manual Baseline Start (min): 10     Manual Baseline End (min): 35
Marker Peak Width (sec): 5    Marker Min Peak Height: 500   Marker Baseline V to V?: N    Marker Baseline V to V pts: 3
Lower Marker Selection: First Peak > 500 RFU                Upper Marker Selection: Last Peak > 500 RFU
Ladder Size (bp): 1, 75, 200, 400, 600, 800, 1000, 1500, 3000, 6000, 10000, 15000, 48500, 200000
Quantification Using: Ladder          Final Concentration (ng/uL): 0.1250          Dilution Factor: 12.0
Min. RFU for Data Processing: 3
```

 Copyright 2015 Advanced Analytical Technologies, Inc.

Sample: 6037.2
Well Location: E5
Created: Thursday, July 21, 2016 3:14:29 PM

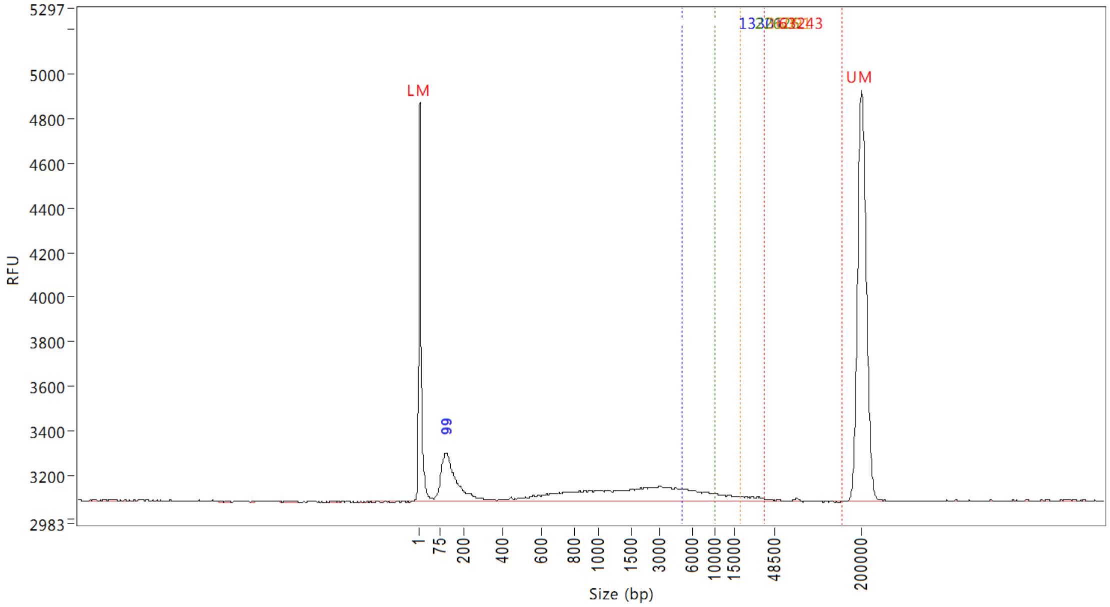

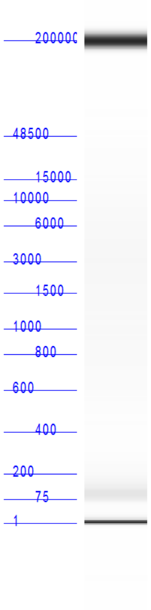

Peak	Size (bp)	Conc. (ng/uL)	From (bp)	To (bp)	Avg. Size (bp)	CV%	RFU	Corr. Peak Area
1	1 (LM)	0.0078	0	46	1	850.87	1787	14.483
2	99	0.0645	46	326	124	42.43	216	10.030
3	200000 (UM)	0.0130	170439	243521	200329	3.59	1839	24.261

TIC:	0.0645	ng/uL
TIM:	1.066	nmole/L
Total Conc.:	0.1715	ng/uL

Smear Analysis						
	40000 bp to 165000 bp	0.0008 ng/ul	0.5 %Total	0.000 nmole/L	63243 Avg. Size (b.p.)	32.56 %CV
	20000 bp to 165000 bp	0.0039 ng/ul	2.3 %Total	0.000 nmole/L	36091 Avg. Size (b.p.)	47.93 %CV
	10000 bp to 165000 bp	0.0094 ng/ul	5.5 %Total	0.001 nmole/L	22626 Avg. Size (b.p.)	70.50 %CV
	5000 bp to 165000 bp	0.0230 ng/ul	13.4 %Total	0.003 nmole/L	13301 Avg. Size (b.p.)	96.42 %CV

```
Sample Peak Width (sec): 10   Sample Min Peak Height: 50    Sample Baseline V to V?: Y    Sample Baseline V to V pts: 3
Sample Filter: Binomial       # of Pts for Filter: 3        Sample Start Region (min): 0  Sample End Region (min): 35
Manual Baseline Start (min): 10     Manual Baseline End (min): 35
Marker Peak Width (sec): 5    Marker Min Peak Height: 500   Marker Baseline V to V?: N    Marker Baseline V to V pts: 3
Lower Marker Selection: First Peak > 500 RFU                Upper Marker Selection: Last Peak > 500 RFU
Ladder Size (bp): 1, 75, 200, 400, 600, 800, 1000, 1500, 3000, 6000, 10000, 15000, 48500, 200000
Quantification Using: Ladder         Final Concentration (ng/uL): 0.1250         Dilution Factor: 12.0
Min. RFU for Data Processing: 3
```

 Copyright 2015 Advanced Analytical Technologies, Inc.

Sample: DNA Size Ladder
Well Location: A12
Created: Thursday, July 21, 2016 3:14:29 PM
Import From: C:\AATI\Saved Ladders\2016 07 21 - HS LF 1 to 12.SCAL
Fit Type: Point to Point

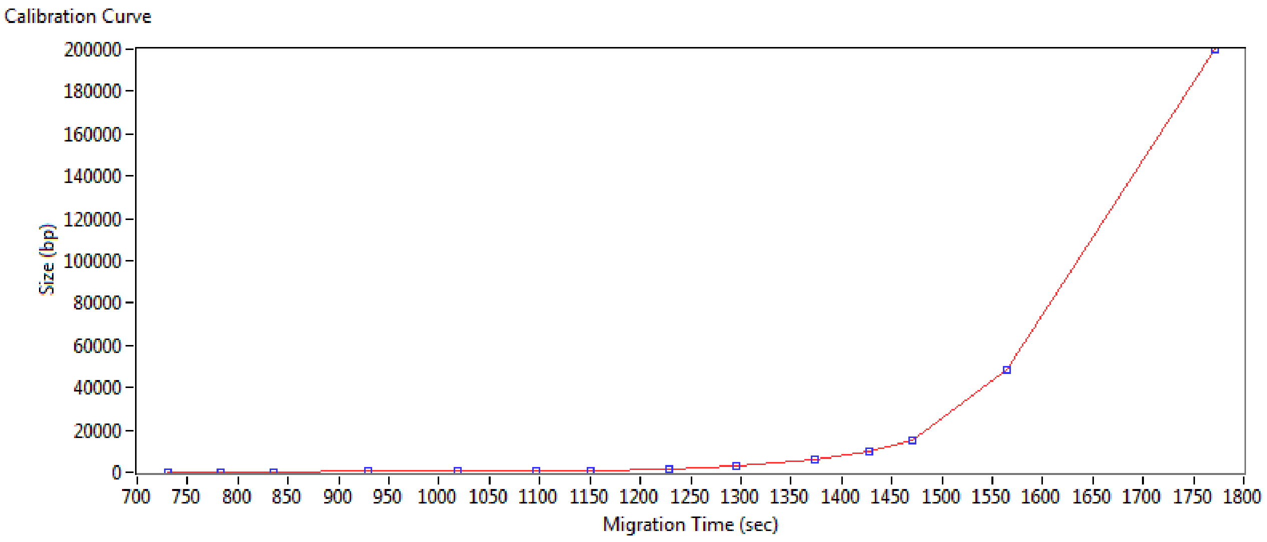

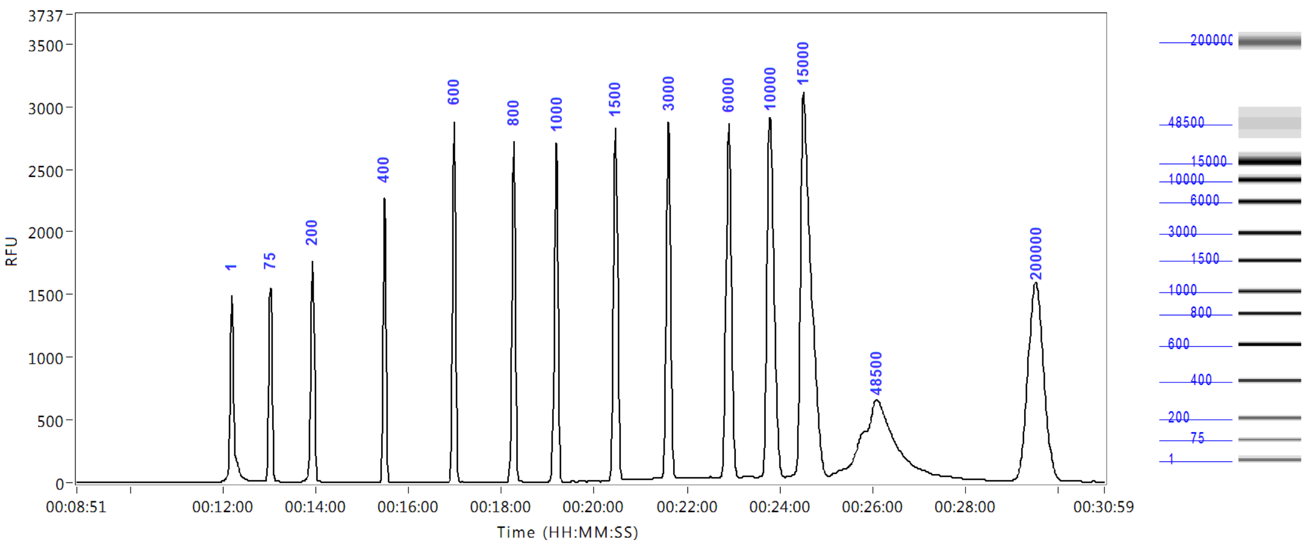

Copyright 2015 Advanced Analytical Technologies, Inc.
